# FARVNet: A Fast and Accurate Range-View-Based Method for Semantic Segmentation of Point Clouds

**DOI:** 10.3390/s25092697

**Published:** 2025-04-24

**Authors:** Chuang Chen, Lulu Zhao, Wenwu Guo, Xia Yuan, Shihan Tan, Jing Hu, Zhenyuan Yang, Shengjie Wang, Wenyi Ge

**Affiliations:** 1 College of Computer Science, Chengdu University of Information Technology, Chengdu 610225, China; 3230609001@stu.cuit.edu.cn (C.C.); 3240609007@stu.cuit.edu.cn (L.Z.); 3230609002@stu.cuit.edu.cn (W.G.); yuanxia@cuit.edu.cn (X.Y.); tsh@cuit.edu.cn (S.T.); lesley123@cuit.edu.cn (J.H.); 2 South West Institute of Technical Physics, Chengdu 610041, China; qujing.ty@163.com; 3 The Key Laboratory of Flight Techniques and Flight Safety, Civil Aviation Flight University of China, Guanghan 618307, China; wang_sheng_jie@126.com; 4 Chengdu Institute of Computer Applications, Chinese Academy of Sciences, Chengdu 610213, China

**Keywords:** 3D point cloud, environmental perception, semantic segmentation, real-time processing, range-view, intensity vanishing state

## Abstract

Environmental perception systems provide foundational geospatial intelligence for precision mapping applications. Light Detection and Ranging (LiDAR) provides critical 3D point cloud data for environmental perception systems, yet efficiently processing unstructured point clouds while extracting semantically meaningful information remains a persistent challenge. This paper presents FARVNet, a novel real-time Range-View (RV)-based semantic segmentation framework that explicitly models the intrinsic correlation between intensity features and spatial coordinates to enhance feature representation in point cloud analysis. Our architecture introduces three key innovations: First, the Geometric Field of View Reconstruction (GFVR) module rectifies spatial distortions and compensates for structural degradation induced during the spherical projection of 3D LiDAR point clouds onto 2D range images. Second, the Intensity Reconstruction (IR) module is employed to update the “Intensity Vanishing State” for zero-intensity points, including those from LiDAR acquisition limitations, thus enhancing the learning ability and robustness of the network. Third, the Adaptive Multi-Scale Feature Fusion (AMSFF) is applied to balance high-frequency and low-frequency features, augmenting the model expressiveness and generalization ability. Experimental evaluations demonstrate that FARVNet achieves state-of-the-art performance in single-sensor real-time segmentation tasks while maintaining computational efficiency suitable for environmental perception systems. Our method ensures high performance while balancing real-time capability, making it highly promising for LiDAR-based real-time applications.

## 1. Introduction

With the rapid development of technologies such as autonomous driving, robot navigation, and unmanned aerial vehicle (UAV) cruising, semantic segmentation has become an indispensable core task in the field of environmental perception [1,2,3,4]. It facilitates timely processing of environmental information and rapid response. Real-time performance and high accuracy are crucial requirements in environmental perception, particularly in complex and dynamically changing scenarios [5]. Balancing fast with high-accuracy semantic segmentation remains a significant challenge [6,7].

As a critical component of environmental perception systems, LiDAR provides essential 3D spatial information [8,9]. The massive, unordered point cloud generated by LiDAR—which uses multi-angle laser beams to measure distances and return signal intensities from target objects—contains rich 3D scene information [10]. Nevertheless, extracting meaningful features and deeper insights from this complex dataset remains a significant challenge. The most significant challenges are threefold: (1) The complexity of the acquired data is exacerbated by variations in acquisition conditions across different devices [8,9,11], resulting in inconsistencies in data quality and significantly increasing the difficulty of data processing and analysis. (2) Real-time, efficient acquisition of discriminative features from 3D spatial data is highly challenging due to the inherent sparsity, unordered nature, and presence of indistinguishable noise [12,13], significantly increasing the complexity of feature learning. (3) Extracting surface features from the spatial relationships within the point cloud is further complicated by the loss of depth information and the complexities introduced by occlusion [14,15]. Therefore, reliable point cloud processing is crucial for practical point cloud applications [16].

Recent research in LiDAR point cloud semantic segmentation has explored a trade-off between accuracy and real-time performance [5]. Methods aiming for high accuracy often utilize more input data (multi-view images, LiDAR point clouds, and color information) and complex, deep network architectures [17,18]. Conversely, methods prioritizing real-time processing employ simpler architectures and computational optimizations to achieve faster speeds, often at the cost of some accuracy. Compared to 3D point cloud processing, 2D feature extraction techniques have made significant progress in various fields [19]. Projecting 3D point cloud data onto a 2D plane significantly enhances computational efficiency and scalability, making it well-suited for real-time applications. However, this dimensionality reduction introduces a substantial trade-off: while computationally advantageous, the resulting loss of information leads to a significant decrease in semantic segmentation accuracy compared to methods utilizing the full 3D data.

Our research aims to design a LiDAR semantic segmentation method that balances efficient inference and high-accuracy prediction, enhancing the adaptability of point cloud perception while improving the robustness of 3D point cloud data and intra-class segmentation consistency in perception scenarios. In summary, our contributions are as follows:

- This work develops a range-view architecture for real-time point cloud semantic segmentation that achieves competitive accuracy with hardware-efficient computation, resolving latency-sensitive precision trade-offs in resource-constrained 3D perception deployments.
- A GFVR module has been implemented to effectively address feature misalignment caused by the projection of 3D space onto a 2D plane.
- An IR module is proposed to update the zero intensity points in the intensity vanishing state, thus mitigating the loss of accuracy due to intensity vanishing.
- The superiority of FARVNet is demonstrated by its accuracy and efficiency on popular benchmark datasets, achieving a balance between high accuracy and real-time performance on both SemanticKITTI [8] and nuScenes [9].

## 2. Related Work

The widespread adoption of semantic segmentation in real-world applications has motivated the development of numerous methods [5,10]. Current mainstream methods primarily include point-based [12,13], voxel-based [20,21], multi-view fusion [22,23], and multi-modal data fusion approaches [17,18,24], all of which have shown significant progress, as shown in Table 1.

### 2.1. Point-Based Methods

Point-based methods that directly process unstructured point clouds face challenges in accurately modeling local geometric structures and capturing long-range contextual dependencies [26,27]. Subsequent research achieved enhanced performance by integrating global features with local feature representations. PointNet [12], a seminal contribution to this field, pioneered the application of Multi-Layer Perceptrons (MLPs) [28] to process points for the extraction of global features. Subsequent studies have proposed innovative architectures that integrate point convolution, graph convolution, and attention mechanisms. RSNet [29] leverages a lightweight Local Dependency Module (LDM) to achieve efficient modeling of local structures within point clouds. To reduce computational complexity in large-scale semantic segmentation, RandLA-Net [13] employs random sampling to decrease point cloud processing time. ECC [30] utilizes spatial graph networks to capture point cloud features and adaptively learns the edge features between neighboring points. DGCNN [31] performs dynamic edge convolution within the neighborhood of each point, and HDGCN [32] uses both depth-wise and pointwise convolutions to extract local and global features. AGCN [33] integrates Self-Attention with Graph Convolutional Networks (GCNs) to model local point relationships and introduces a global point graph to capture the relative positional information of each point. However, due to the limitation of point cloud quantity, point-based methods are still unable to process large-scale point clouds in real time.

### 2.2. Voxel-Based Methods

These methods typically treat voxelized point clouds as 3D grids, applying 3D Convolution Neural Networks (CNNs) to extract features from the volumetric representation. ShapeNets [34] employs a convolutional deep belief network to model the probabilistic distribution of 3D shapes, thereby constructing a hierarchical representation in a bottom-up fashion. The computational complexity of this method scales cubically with voxel size, posing a significant challenge for processing high-resolution point clouds. Specifically, to preserve fine geometric details, smaller voxels are required, which in turn increase computational overhead, whereas larger voxels result in a loss of detail. OctNet [35] utilizes octree decomposition to effectively handle sparsely distributed point clouds, reducing both data size and computational complexity, while facilitating a rapid nearest-neighbor search. PointGrid [36] utilizes a combined point and grid representation, employing a fixed number of points to extract geometric detail features and improve model efficiency. However, these methods still demand significant computational and memory resources, rendering them unsuitable for resource-constrained edge devices.

### 2.3. Multi-Modal Data Fusion Methods

These methods integrate data from multiple modal and diverse data types, yielding more comprehensive feature information than single sources. TransFusion [37] seamlessly integrates images and point clouds to fully leverage spatial structural information. UniSeg [18] fuses voxel, view, and image features to fully utilize multi-modal semantic information. 2DPASS [24] employs auxiliary modality fusion and multi-scale feature fusion to extract richer semantic structural information from multi-modal data. Real-time multi-modal data fusion faces significant challenges due to the complexity and volume of data from diverse sources [20]. These sources often have varying formats, update rates, and sensor characteristics, creating integration difficulties and leading to processing delays and computational bottlenecks. This hinders the ability to meet real-time demands in dynamic environments.

### 2.4. Multi-View Fusion Methods

Multi-view fusion methods leverage the complementary information obtained from different viewpoints of the same point cloud data, for example, BEV and RV,df to improve model accuracy and robustness in semantic segmentation [23]. MVCNN [25] leverages a multi-view CNN architecture trained on multiple rendered 2D images, employing max pooling to aggregate a global feature representation from these diverse perspectives. AMVNet [22] uses RV and BEV, each for point cloud feature extraction. For uncertain point clouds, neighbourhood features are fused to predict the final category. CFNet [23] uses Center Focusing Feature Encoding (CFFE) to mitigate viewpoint misalignment. Overall, features between different viewpoint branches require additional precise alignment and fusion, with delays in real-time performance.

### 2.5. Range-View Methods

RV is widely used in semantic segmentation and point cloud upsampling due to its effectiveness and real-time capabilities [38,39,40]. TFNet [41] leverages temporal sequences to address the “many-to-one” projection issue and integrates a voting mechanism to correct misclassified labels. However, the voting strategy struggles to distinguish boundary regions. FRNet [5], which directly utilizes raw intensity, fails to effectively identify regions with intensity vanishing. FIDNet [42] uses bilinear interpolation to fuse multi-resolution features, but it fails to distinguish complex spatial relationships.

Compared to state-of-the-art techniques, RV often exhibits suboptimal performance, primarily due to the inherent challenges in learning 3D spatial features from projected range images, which are prone to various forms of interference. (1) The 3D point cloud data are sparse and unordered [43,44]: limitations in the original range image, such as distortions, occlusions, and incomplete feature representations, directly compromise the network backbone capacity for accurate spatial feature extraction. To address this, we propose the GFVR module, which enhances image quality and compensates for performance degradation caused by point cloud misalignment. (2) Intensity measurements are susceptible to significant noise and variation: as the distance between the sensor and the target object increases, intensity generally exhibits an attenuating trend accompanied by fluctuations. This attenuation is a consequence of laser beam propagation losses, and intensity is further modulated by various factors, including target surface reflectivity and material properties. Accurate discrimination between intensity-dropout states and normal point clouds during range image projection is critical for enhancing system robustness. We address this limitation by introducing an IR module, which corrects pseudo-intensity-dropout artifacts effectively. (3) Image boundaries are blurred: RV-based methods typically generate 2D feature maps with poorly defined object boundaries, resulting in intra-class inconsistencies in the segmentation outcomes. The application of AMSFF learns the balance between latent high-level and low-level features, enhancing the system’s robustness.

## 3. Method

**Figure 1:**
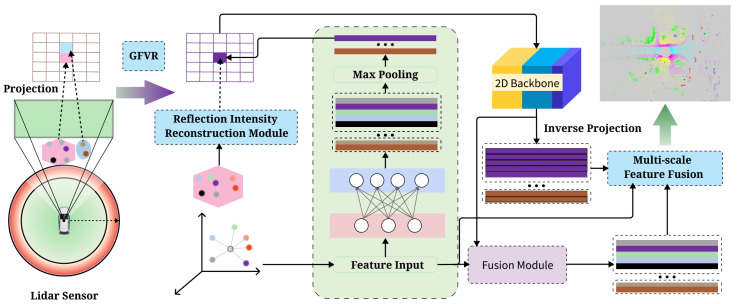
FARVNet overview. It consists of three steps: (1) The feature encoding incorporates the Geometric Field of View Reconstruction (GFVR) and Intensity Reconstruction (IR) modules. (2) The 2D backbone network extracts multi-scale features. (3) The Adaptive Multi-Scale Feature Fusion (AMSFF) of hierarchically extracted features predicts the final class label.

This section provide a detailed explanation of the FARVNet (Section 3.1). The architecture overview of the proposed FARVNet framework is depicted in Figure 1.

### 3.1. Overview

As shown in Figure 1, the network architecture is primarily divided into three parts: feature encoding is performed to deeply integrate 3D spatial coordinates and intensity features, with the IR and GFVR modules; feature extraction is performed to extract multi-scale features; Adaptive Multi-Scale Feature Fusion is used to integrate high-dimensional and low-dimensional features.

#### 3.1.1. Feature Encoding

**Figure 2:**
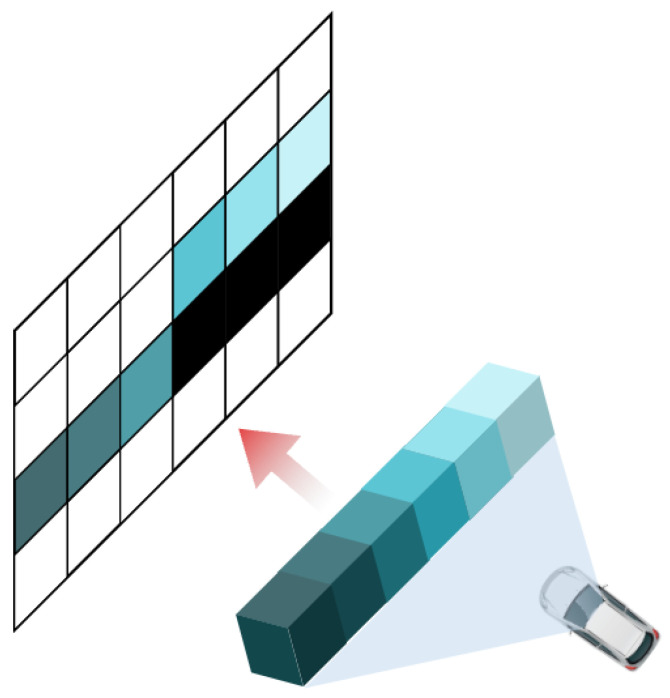
Projection offset caused by floating-point rounding during the projection process. Black represents pixel voids caused by projection offset.

**Figure 3:**
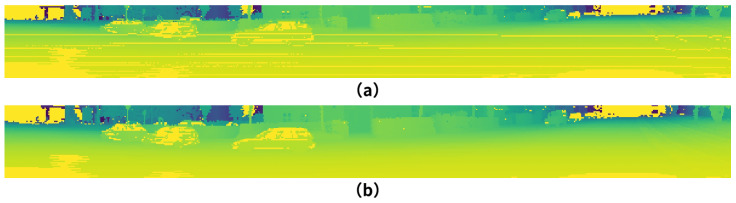
Comparison of range projection methods: (**a**) typical method described in Equation (A1), (**b**) our GFVR-based method in Equation (1). Our approach clearly outperforms traditional projection rules.

It is noteworthy that, with the development of hardware devices, a growing diversity of LiDAR acquisition devices has emerged, leading to increasingly rich data [11,45]. These devices exhibit variations in key parameters such as the number of laser beams, effective range, field of view (FOV), and intensity distribution. Beyond the inherent differences in LiDAR sensor specifications, point cloud scene acquisition is also influenced by the platform on which the sensor is mounted. For instance, increasing platform altitude necessitates adjustments to the FOV and azimuth angle to effectively capture surrounding environmental information. Additionally, its accuracy is also affected by positional offset, as shown in Figure 2, which leads to the clustering of point clouds and the formation of numerous voids, as shown in Figure 3, at the top. The detailed formulation can be found in Appendix A.

We employ GFVR that adjusts for the distance discrepancies between the 3D point cloud and the true origin, as shown in Equation (1):

(1) $$GFVR=\left\{\begin{array}{c} \Delta =Offset(arcsin\left(P_{n}^{z},{\left(P_{n}^{d}\right)}^{-1}\right),\phi ,Envs)\\ {\tilde{P}}_{n}^{d}=\sqrt{{\left(P_{n}^{x}\right)}^{2}+{\left(P_{n}^{y}\right)}^{2}+{\left(P_{n}^{z}+\Delta \right)}^{2}}\\ {\tilde{v}}_{n}=\left[1-\left(arcsin\left(P_{n}^{z}+\Delta ,{\left({\tilde{P}}_{n}^{d}\right)}^{-1}\right)+{\tilde{\varphi }}_{down}\right){\tilde{f}}^{-1}\right]H\\ u_{n}=\frac{1}{2}\left[1-arctan\left(P_{n}^{y},P_{n}^{x}\right)\pi^{-1}\right]W \end{array}\right.$$

The Offset calculates the vertical offset for each laser beam point cloud using weighting parameters, ϕ represents the FOV angle range of the radar device, Envs represents the LiDAR sensor’s own height and its spatial position relative to the ground, and $\left(P_{n}^{x},P_{n}^{y},P_{n}^{z}\right)$ represents the 3D spatial coordinates of a point cloud, while constraints within the FOV are simultaneously applied to prevent feature loss by ${\tilde{\varphi }}_{down}$ and ${\tilde{f}}^{-1}$, as shown in Figure 3, at the bottom. First, the point cloud is partitioned into H regions. Then, the overall offset for each region is calculated through the regional partitioning. To maintain the continuity and integrity of the point cloud scene, the offsets are smoothed to eliminate potential discontinuities or abrupt changes.

GFVR partitions point cloud scene P into K projection spaces, with projection performed using Equation (1): $P=\{P_{1},P_{2},\ldots ,P_{K}\}$. $P_{K}\in R^{m\times c}$. Each projection space contains a varying number of points *m*, and each point has the same feature dimensionality *c*.

(2) $$P_{1},P_{2},\ldots ,P_{K}=GFVR\left(P\right)$$

**Figure 4:**
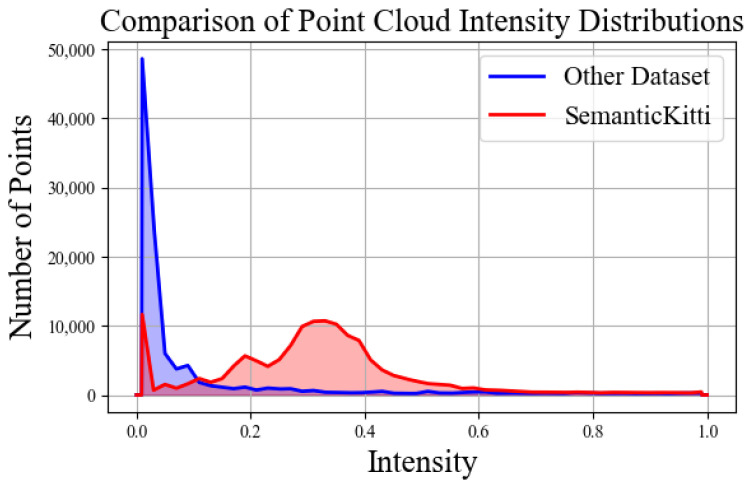
Comparison of intensity distribution between the SemanticKITTI [8] scenario (red) and a domestic point cloud acquisition device (blue).

Point cloud intensity I∈[0,1], reflecting the ability of the object surface to reflect laser pulses, and is closely related to factors such as the material of the object, surface roughness, and relative orientation. However, in practice, there is a special phenomenon of intensity drop-out, which is represented by an intensity value of zero. This typically indicates that the LiDAR system failed to receive a reflected signal from the target object. Potential causes include the target object’s material properties, surface roughness, the distance between the object and the LiDAR, the angle of incidence, LiDAR positioning errors, or even equipment malfunction, as illustrated in Figure 4.

**Figure 5:**
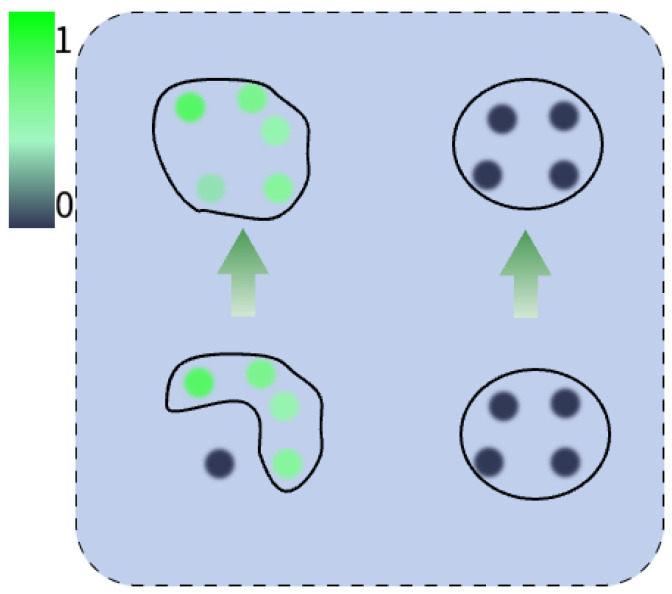
Pseudo-noise in the state of vanishing intensity is updated by intensity perception (**left**), and a generally low intensity is considered to be caused by the object (**right**).

Specifically, to prevent the neural network from overfitting to these outliers, employing an outlier detection mechanism is an effective strategy. The point cloud scene is partitioned into *m* project space based on GFVR. This clustering process incorporates both distance and intensity awareness, as shown in Figure 5. Mean pooling is then applied to the intensity values of the point cloud data within each projected spatial region, as shown in Equation (3).

(3) $$IR=\left\{\begin{array}{cc} \frac{\sum_{i=0}^{n}I_{i}}{\sum_{i=0}^{n}1_{\left\{I_{i}\neq 0\right\}}} & \sum_{i=0}^{n}1_{\left\{I_{i}\neq 0\right\}}\geq \sum_{i=0}^{n}1_{\left\{I_{i}=0\right\}}\\ \min_{i\in n}I_{i}\neq 0 & \sum_{i=0}^{n}1_{\left\{I_{i}\neq 0\right\}}<\sum_{i=0}^{n}1_{\left\{I_{i}=0\right\}}\\ 0 & \sum_{i=0}^{n}1_{\left\{I_{i}=0\right\}}=n \end{array}\right.$$

(4) $$\left\{{\bar{P}}_{1},{\bar{P}}_{2},\ldots ,{\bar{P}}_{K}\right\}=IR\left(P_{1},P_{2},\ldots ,P_{K}\right)$$

where ∑ denotes the summation of values, and *I* represents the intensity value of a single point in the projection space. The content inside the brackets represents the condition.

Next, global features $F_{p}\in R^{m\times C}$ from the point cloud are efficiently learned using MLP [28]. Feature dimensionality reduction and max pooling are then applied to each projection space, yielding projection space features $F_{ps}\in R^{1\times D}$, features shared by all points within a projection space. These features are finally projected to 2D range image features $F_{ri}\in R^{B\times D\times H\times W}$. Here, *D* represents the output dimensionality of the MLP, and *B* represents the batch size:

(5) $$\left\{\begin{array}{c} F_{p}=MLP\left(\left\{{\bar{P}}_{1},{\bar{P}}_{2},\ldots ,{\bar{P}}_{K}\right\}\right)\\ F_{ps}=MAXPooling\left(F_{p}\right)\\ F_{ri}=Project\left(F_{ps}\right) \end{array}\right.$$

Input feature dimensionality expanded from 4 to 10, ${\bar{P}}_{k}\in R^{m\times 10}$. Expanded features include initial 3D coordinates and intensity ($P_{n}^{x}$, $P_{n}^{y}$, $P_{n}^{z}$, $P_{n}^{I}$) of the point cloud. Manhattan distance, vector difference, and intensity change between each point in the projection space and the interval virtual center are as follows: ($P_{n}^{x}-\bar{P_{m}^{x}}$, $P_{n}^{y}-\bar{P_{m}^{y}}$, $P_{n}^{z}-\bar{P_{m}^{z}}$, Dd, $P_{n}^{I}-\bar{P_{m}^{I}}$). Each point cloud depth is represented by $P_{n}^{d}$.

#### 3.1.2. Feature Extraction

The framework utilizes cascaded residual convolution blocks to construct a pyramidal 2D backbone that hierarchically generates multi-scale feature representations. At each feature extraction stage, per-point features within the projection space are iteratively updated. These updated per-point features then update the projection space features.

Feeding $F_{ri}^{i-1}$ into a residual network yields $F_{ri}^{i}$, a high-level feature representation capturing both local and global spatial contexts within the 2D image. This network is designed to extract multi-resolution features across different scales and increase channel depth for enhanced feature diversity, ultimately improving performance on complex tasks:

(6) $$F_{ri}^{i}=ResBlock\left(F_{ri}^{i-1}\right)$$

The range image constitutes a geometric abstraction of the 3D environment rather than an isomorphic scene representation. The naive application of 2D convolutional features for 3D semantic inference remains intrinsically constrained by representational disparity across dimensional domains. To maintain consistency between point and projection space features, feature vectors extracted from both point cloud and image spaces are concatenated. This fused feature vector is then input to an MLP for non-linear transformation and feature fusion:

(7) $$\tilde{F_{p}^{i}}=MLP\left(Inproject\left(F_{ri}^{i}\right),F_{p}^{i-1}\right)$$

Then, the updated point features are projected back to the range image, and image features are fused using convolution. RVFusion comprises three main components: first, a 2D convolutional layer with a 3 × 3 kernel and a stride of 1 for local feature extraction; second, a fully connected layer that maps the extracted features to a specified dimensional space; and finally, an activation layer that further enhances the expressiveness of the features, thereby achieving efficient feature fusion:

(8) $$\tilde{F_{ri}^{i}}=RVFusion\left(Project\left(\tilde{F_{p}^{i}}\right),F_{ri}^{i}\right)$$

Finally, range image features are updated using a residual attention weighting mechanism. This residual attention mechanism effectively strengthens important features while suppressing irrelevant information, improving feature expressiveness:

(9) $$F_{ri}^{i}=\tilde{F_{ri}^{i}}\times Attention\left(\tilde{F_{ri}^{i}}\right)+F_{ri}^{i}$$

#### 3.1.3. Adaptive Multi-Scale Feature Fusion Module

Inspired by FreqFusion [46], cross-resolution feature aggregation via frequency-aware attention mechanisms in hierarchical neural representations. Ours employs an Adaptive Low-Pass Filter (ALPF) generator, an offset generator, and an Adaptive High-Pass Filter (AHPF) generator to fuse high-level and low-level features. An ALPF generator predicts spatially varying low-pass filters to attenuate high-frequency components within objects, reducing intra-class inconsistencies during upsampling. An Offset generator refines large inconsistencies and thin boundaries by replacing inconsistent features with more consistent ones through resampling. An AHPF generator enhances the high-frequency detail and boundary information lost during the downsampling process:

(10) $$\left\{\begin{array}{c} {\tilde{Y}}_{u,v}^{i+1}=UPSamping\left(ALPH\left(Y_{u,v}^{i+1}\right)\right)\\ {\tilde{X}}_{u,v}^{i}=AHPF\left(X_{u,v}^{i}\right)+X_{u,v}^{i}\\ Y_{u,v}^{i}={\tilde{Y}}_{u+\alpha ,v+\beta }^{i+1}+{\tilde{X}}_{u,v}^{i} \end{array}\right.$$

where $Y_{u,v}^{i+1}\in R^{D\times h\times w}$, and $X_{u,v}^{i}\in R^{D\times 2h\times 2w}$ represent the *i*-th features generated by the backbone and the fused feature at the *i*-th level, respectively. Offset values (α, β) are predicted by the offset generator for the feature located at (*u*, *v*).

## 4. Experiments

This section demonstrates the robustness of FARVNet as well as the balance between accuracy and efficiency. We begin by describing the benchmark datasets and hyperparameters. Experimental results demonstrate that our method achieves an optimal balance between efficiency and accuracy, and we further validate its performance and efficiency on specialized devices. Finally, ablation studies are conducted to analyze the contribution of each component.

### 4.1. Datasets

We conducted comprehensive evaluations on two widely used datasets. SemanticKITTI [8] is a large-scale outdoor autonomous driving LiDAR dataset collected in Karlsruhe, Germany, using a Velodyne HDL-64E LiDAR sensor. It consists of 22 sequences, with Sequences 0–7 and 9–10 containing point clouds and labels for training, Sequence 8 for validation, and Sequences 11–21 for online testing. Each scene contains approximately 120 thousands points. The original data uses 28 classes to categorize the entire scene, with a vertical field of view of [−25, 3] degrees. nuScenes [9] is a widely used autonomous driving dataset collected using a 32-beam LiDAR sensor. This dataset contains 1000 driving scenes with dense point clouds, annotated with 32 classes and evaluated using 16 semantic classes, with a vertical field of view of [−30, 10] degrees.

### 4.2. Implementation Details

Our FARVNet framework is built upon the widely adopted MMDetection3D [47] platform. The architecture employs a customized ResNet34 [19] variant as its 2D backbone network, with feature map dimensions specifically configured for different datasets: 64 × 512 pixels for SemanticKITTI [8] and 32×480 pixels for nuScenes [9]. The optimization strategy utilizes the AdamW [48] optimizer initialized with a base learning rate of 0.001, complemented by the OneCycle [49] learning rate policy to achieve adaptive rate scheduling throughout the training phase. All experiments were conducted with a mini-batch size of 4 to balance computational efficiency and memory constraints.

## 5. Results

### 5.1. Quantitative Results

Table 2 and Table 3 present a comparison of our method with existing state-of-the-art methods on the SemanticKITTI [8] and nuScenes [9] val sets. The results demonstrate that FARVNet significantly outperforms previous approaches on most metrics, exhibiting superior performance. Specifically, on the SemanticKITTI [8] val set, our method surpasses the recently proposed FRNet [5] by +1.0 mIoU. On the nuScenes [9] val set, our method outperforms the recently proposed WaffleAndRange [50] by +0.7 mIoU.

### 5.2. Qualitative Results

**Figure 6:**
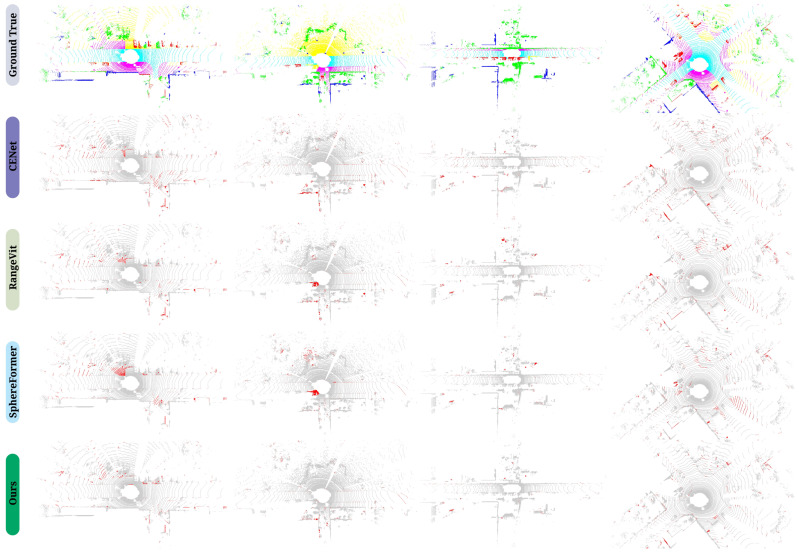
Visualization of multiple real-time semantic segmentation methods based on RV and using the SemanticKITTI [8] val set, with gray representing correct labels and red representing incorrect labels.

**Figure 7:**
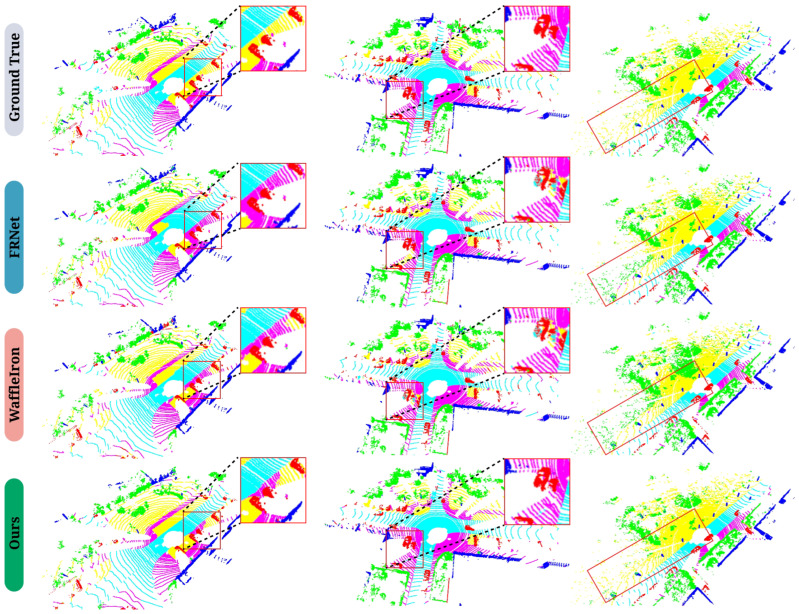
Visual comparison of high-level semantic segmentation network model performance on SemanticKITTI [8] Sequence 08, with magnified views highlighting key differences. Ground True indicates the true labels.

**Figure 8:**
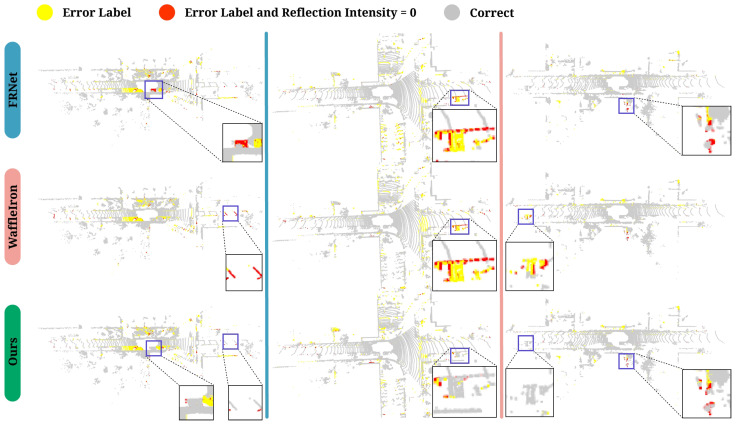
Comparison of different semantic segmentation network ability to handle intensity drop-out points. Yellow indicates error class segmentation; red indicates incorrect segmentation where the point cloud intensity = 0.

Visualization examples of real-time semantic segmentation on SemanticKITTI [8] Sequence 08, based on RV and high-performance LiDAR, are provided. Figure 6 shows the Ground Truth (GT) and segmentation results from several methods. Our method exhibits the fewest mis-segmented regions, demonstrating higher accuracy and robustness. This further validates the effectiveness of our method in handling complex scenes, enabling better identification and distinction between object classes. Figure 7 clearly illustrates that existing real-time semantic segmentation methods struggle with sparse boundary regions, resulting in incomplete and mis-segmented areas, such as sidewalks and vegetation. In contrast, the proposed FARVNet method demonstrates the most faithful segmentation alignment with ground truth values. Furthermore, our method shows a significant advantage in handling low-intensity regions. As shown in Figure 8, our method demonstrates strong competitiveness in both long-distance and short-distance scenarios.

### 5.3. Runtime and Model Parameters

**Figure 9:**
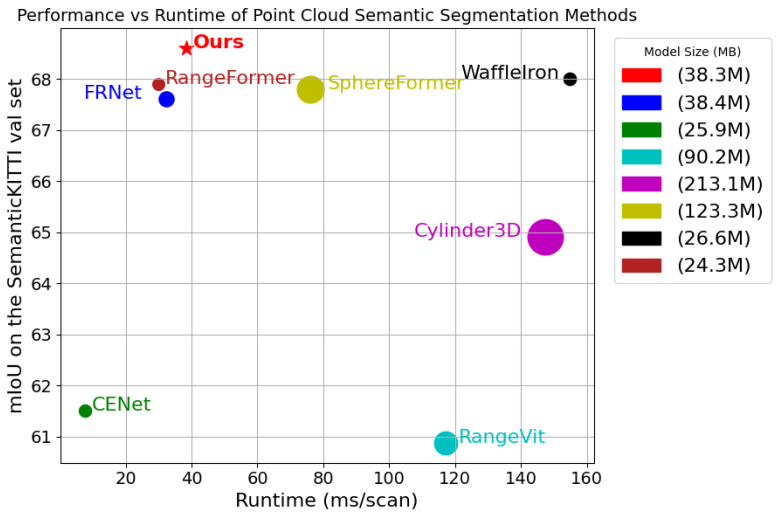
mIoU vs. runtime on the SemanticKITTI [8] val set. The size of the markers represents the model’s number of parameters. Runtime measurements are taken on a single NVIDIA RTX 4090 GPU.

Runtime was also evaluated using the SemanticKITTI [8] val set. The runtime for all methods was measured on a single NVIDIA RTX 4090 GPU without any acceleration techniques, as shown in Figure 9. Our method is end-to-end and requires no additional computation time. Both the GFVR and IR have linear time complexity, ensuring efficient processing. The inference time is 38.5 ms, 1.98 times faster than SphereFormer [61] and 4 times faster than WaffleIron [62]. Meanwhile, our method adopts a more lightweight design, maintaining high performance while having fewer parameters, and the inference process requires only approximately 1500 MB of GPU memory.

### 5.4. Ablation Study

Ablative analyses were conducted on the SemanticKITTI [8] val set and nuScenes [9] val set with the above experimental setup. As shown in Table 4, we validated the effectiveness of the MSFF, GFVR, IR, and TTA modules by incorporating different modules, and compared quantitative metrics with the baseline model. In particular, the MSFF, GFVR, IR modules improve 1.93% in mIoU and 1.89% in mAcc on SemanticKITTI [8], and 1.18% in mIoU and 0.5% in mAcc on nuScenes [9], compared to baseline. The underlying reason for this performance difference lies in the high-quality range image and stronger pseudo-noise recognition. Further study of the impact of different IR algorithms on accuracy is shown in Table 5. The mean pooling operation exhibits significant advantages in feature representation over others.

### 5.5. Failure Cases

**Figure 10:**
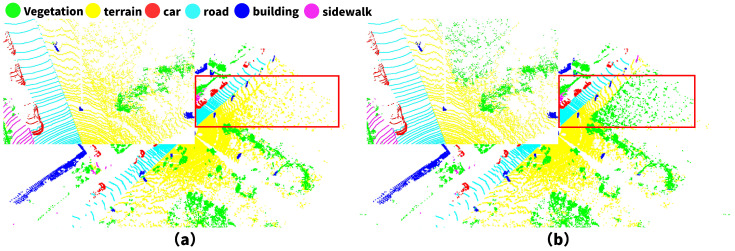
Semantic segmentation failure in complex terrain, as illustrated by the real-world segmentation result in (**a**) and the result from our method in (**b**). The similar spatial distribution and elevation features, along with point cloud sparsity, make it difficult for the model to distinguish between “vegetation” and “terrain”.

Although FARVNet outperforms state-of-the-art methods in multiple metrics, its semantic segmentation accuracy remains limited in specific scenarios. As illustrated in Figure 10, the model struggles to effectively distinguish low-lying vegetation from uneven ground surfaces, resulting in misclassification between the “vegetation” and “terrain” labels.

## 6. Discussion

As quantitatively demonstrated through the comparative analysis in Table 2 and Table 3, the conventional projection formula assumes point clouds originating from a single coordinate system, utilizing 3D spatial coordinates to compute azimuth and elevation angles. However, since laser beams capturing scene information are not emitted from identical points, the displacement discrepancy between laser beams and physical objects generates extensive void pixels in the range image. The proposed FARVNet method utilizes the GFVR module to mitigate laser beam displacement deviations, thereby improving range image quality and enabling more comprehensive scene feature learning, as shown in Figure 6.

FRNet [5] and WaffleIron [62] achieve multi-scale feature fusion solely through simple linear interpolation upsampling operations. Lu [68] found that simple linear interpolation resulted in over-smoothing, leading to boundary displacement and significantly impacting the quality of the inverse mapping from the distance view to 3D space, as shown in the experimental results in Figure 7. In contrast, the AMSFF module balances high-frequency and low-frequency features through adaptive frequency perception, reducing intra-class inconsistencies in multi-scale fusion, effectively distinguishing intra-class features, maintaining intra-class consistency, and avoiding incomplete segmentation.

Figure 8 shows the impact of intensity on the model’s accuracy. The current mainstream methods recognize the importance of intensity, but they do not consider its instability. The intensity drop-out phenomenon presents a significant challenge for point cloud data processing, as network models struggle to accurately distinguish these anomalous intensity values from valid data. Specifically, the model does not effectively differentiate noise from meaningful signals, leading to overfitting, particularly in point cloud scenes containing substantial noise. Therefore, the IR module effectively manages these outliers and mitigates their negative impact on network training, which is essential for enhancing model performance.

In sparse scenes, severe loss of spatial geometric information caused by projection—particularly at large-area sparse junctions of terrain—results in chaotic label segmentation, as illustrated in Figure 10.

## 7. Considerations for Future Work

This study proposes a novel range-image-based point cloud semantic segmentation method that enhances distance image quality, deeply explores the intrinsic correlation between spatial coordinates and intensity information, balances intra-class inconsistency between high-dimensional and low-dimensional features, and improves model accuracy and robustness.

A potential limitation of our method is its reliance on the spatial coordinates and intensity of the point cloud, which limits its performance under sparse point cloud conditions. Although the method shows significant improvement on 64-line and 32-line datasets, it still exhibits drawbacks in sparse regions at long distances. Future work will further investigate the network’s performance in sparse and challenging environments, with a focus on enhancing accuracy in distant sparse regions through long-range dependency modeling.

## Figures and Tables

**Table 1:** Comparisons among different LiDAR representations.

Based	Input	Representative
Raw Point	Point	PointNet [12], RandLA-Net [13]
Voxel	Point	PVCNN [20], PVRCNN++ [21]
Multi-Modal	Point+Image	FuseSeg [17], Uniseg [18]
Multi-View	Point	AMVNet [22], CFNet [23], MVCNN [25]
Range-View	Point	RangeFormer [6], FRNet [5], RangePerception [7]

**Table 2:** The class-wise IoU scores of different LiDAR semantic segmentation approaches on the SemanticKITTI [8] val set. All mIoU scores are given in percentage (%). The **best** and second best scores for each class are highlighted in **bold** and underline.

Method	mIoU	Car	Bicycle	Motorcycle	Truck	Other-vehicle	Person	Bicyclist	Motorcyclist	Road	Parking	Sidewalk	Other-ground	Building	Fence	Vegetation	Trunk	Terrain	Pole	Traffic-sign
RandLA-Net [13]	50.0	92.0	8.0	12.8	74.8	46.7	52.3	46.0	0.0	93.4	32.7	73.4	0.1	84.0	43.5	83.7	57.3	73.1	48.0	27.0
RangeNet++ [51]	51.0	89.4	26.5	48.4	33.9	26.7	54.8	69.4	0.0	92.9	37.0	69.9	0.0	83.4	51.0	83.3	54.0	68.1	49.8	34.0
SequeezeSegV2 [52]	40.8	82.7	15.1	22.7	25.6	26.9	22.9	44.5	0.0	92.7	39.7	70.7	0.1	71.6	37.0	74.6	35.8	68.1	21.8	22.2
SequeezeSegV3 [53]	53.3	87.1	34.3	48.6	47.5	47.1	58.1	53.8	0.0	95.3	43.1	78.2	0.3	78.9	53.2	82.3	55.5	70.4	46.3	33.2
SalasNet [54]	59.4	90.5	44.6	49.6	86.3	54.6	74.0	81.4	0.0	93.4	40.6	69.1	0.0	84.6	53.0	83.6	64.3	64.2	54.4	39.8
MinkowskiNet [55]	58.5	95.0	23.9	50.4	55.3	45.9	65.6	82.2	0.0	94.3	43.7	76.4	0.0	87.9	57.6	87.4	67.7	71.5	63.5	43.6
SPVNAS [56]	62.3	96.5	44.8	63.1	55.9	64.3	72.0	86.0	0.0	93.9	42.4	75.9	0.0	88.8	59.1	88.0	67.5	73.0	63.5	44.3
Cylinder3D [57]	64.9	96.4	61.5	$\underline{78.2}$	66.3	69.8	$\underline{80.8}$	$\underline{93.3}$	0.0	94.9	41.5	78.0	1.4	87.5	55.0	86.7	72.2	68.8	63.0	42.1
PMF [58]	63.9	95.4	47.8	62.9	68.4	75.2	78.9	71.6	0.0	96.4	43.5	80.5	1.0	88.7	60.1	$\underline{88.6}$	$\underline{72.7}$	$\underline{75.3}$	$\underline{65.5}$	43.0
rangvit [59]	60.9	94.7	44.1	61.4	71.9	37.7	65.3	75.5	0.0	95.5	48.4	83.1	0.0	88.3	60.0	86.3	65.3	72.7	63.1	42.7
CENet [60]	61.5	91.6	42.4	61.7	82.4	63.5	64.4	76.6	0.0	93.0	50.3	72.7	0.1	85.0	54.4	84.1	61.0	70.3	55.2	42.8
RangeFormer [6]	66.5	95.0	58.1	72.1	85.1	59.8	76.9	86.4	0.2	94.8	$\underline{55.5}$	81.7	$\underline{13.0}$	88.5	64.5	86.5	66.8	73.0	64.0	52.0
SphereFormer [61]	67.8	96.8	51.0	75.0	93.4	64.4	77.0	92.6	$\underline{0.8}$	94.7	53.2	52.1	3.7	$\underline{90.7}$	58.5	88.7	71.3	75.9	64.7	54.5
FRNet [5]	67.6	$\underline{97.2}$	53.3	72.9	81.5	72.9	77.2	90.8	0.2	95.9	53.7	$\underline{83.9}$	9.0	90.5	$\underline{65.9}$	87.0	66.8	72.6	64.0	47.9
waffleIron [62]	$\underline{68.0}$	96.1	$\underline{58.1}$	79.7	77.4	59.0	81.1	92.2	1.3	95.5	50.2	83.6	6.0	92.1	67.5	87.8	73.8	73.0	65.7	$\underline{52.2}$
FARVNet	69.4	97.2	56.5	77.1	$\underline{90.2}$	$\underline{72.9}$	78.6	93.9	0.0	$\underline{96.0}$	57.8	84.2	21.4	90.0	62.4	87.2	66.5	73.2	64.8	48.1

**Table 3:** The class-wise IoU scores of different LiDAR semantic segmentation approaches on the *val* set of nuScenes [9]. All IoU scores are given in percentage (%). The **best** and second best scores for each class are highlighted in **bold** and underline.

Method	mIoU	Barrier	Bicycle	Bus	Car	Construction-vehicle	Motorcycle	Pedestrian	Traffic-cone	Trailer	Truck	Driveable-surface	Other-ground	Sidewalk	Terrain	Manmade	Vegetation
AF2S3Net [63]	62.2	60.3	12.6	82.3	80.0	20.1	62.0	59.0	49.0	42.2	67.4	94.2	68.0	64.1	68.6	82.9	82.4
RangeNet++ [51]	65.5	66.0	21.3	77.2	80.9	30.2	66.8	69.6	52.1	54.2	72.3	94.1	66.6	63.5	70.1	83.1	79.8
PolarNet [64]	71.0	74.7	28.2	85.3	90.9	35.1	77.5	71.3	58.8	57.4	76.1	96.5	71.1	74.7	74.0	87.3	85.7
PCSCNet [65]	72.0	73.3	42.2	87.8	86.1	44.9	82.2	76.1	62.9	49.3	77.3	95.2	66.9	69.5	72.3	83.7	82.5
SalsaNext [54]	72.2	74.8	34.1	85.9	88.4	42.2	72.4	72.2	63.1	61.3	76.5	96.0	70.8	71.2	71.5	86.7	84.4
SVASeg [66]	74.7	73.1	44.5	88.4	86.6	48.2	80.5	77.7	65.6	57.5	82.1	96.5	70.5	74.7	74.6	87.3	86.9
RangeViT [59]	75.2	75.5	40.7	88.3	90.1	49.3	79.3	77.2	66.3	65.2	80.0	96.4	71.4	73.8	73.8	89.9	87.2
Cylinder3D [57]	76.1	76.4	40.3	91.2	93.8	51.3	78.0	78.9	64.9	62.1	84.4	96.8	71.6	76.4	$\underline{75.4}$	90.5	87.4
AMVNet [22]	76.1	79.8	32.4	82.2	86.4	62.5	81.9	75.3	72.3	83.5	65.1	97.4	67.0	78.8	74.6	90.8	87.9
RPVNet [67]	77.6	78.2	43.4	92.7	93.2	49.0	$\underline{85.7}$	80.5	66.0	66.9	84.0	96.9	73.5	75.9	70.6	90.6	88.9
WaffleIron [62]	77.6	$\underline{78.7}$	51.3	93.6	88.2	47.2	86.5	81.7	68.9	69.3	83.1	96.9	74.3	75.6	74.2	87.2	85.2
RangeFormer [6]	78.1	78.0	45.2	94.0	92.9	58.7	83.9	77.9	69.1	63.7	85.6	96.7	74.5	75.1	75.3	89.1	87.5
SphereFormer [61]	78.4	77.7	43.8	$\underline{94.5}$	93.1	52.4	86.9	$\underline{81.2}$	65.4	73.4	$\underline{85.3}$	97.0	73.4	75.4	75.0	91.0	89.2
WaffleAndRange [50]	77.6	78.5	$\underline{49.6}$	91.8	87.6	52.7	86.7	82.2	$\underline{70.1}$	67.2	79.7	97.0	$\underline{74.7}$	$\underline{76.8}$	74.9	87.5	85.0
FARVNet	$\underline{78.3}$	78.3	42.5	95.5	91.8	$\underline{58.9}$	84.4	77.7	67.5	68.9	83.5	$\underline{97.0}$	77.2	76.1	76.0	89.2	87.5

**Table 4:** Ablation study of each component in Ours on the valset of SemanticKITTI [8] and nuScenes [9]. BL: BaseLine. GFVR: Geometric Field of View Reconstruction. IR: Intensity Reconstruction. TTA: Test Time Augmentation. All mIoU and mAcc scores are given in percentage (%).

BL	MSFF	GFVR	IR	TTA	SemKITTI		nuScenes	
					**mIoU**	**mAcc**	**mIoU**	**mAcc**
√	√				67.3	74.0	76.1	83.9
√	√	√			67.8	75.3	76.3	83.9
√	√	√	√		68.6	75.4	77.0	84.4
√	√	√	√	√	69.4	75.5	78.3	85.0

**Table 5:** Impact of different IR algorithms on the mIoU metric.

Mean	Max	Min
+0.8	+0.1	+0.2

## Data Availability

Publicly available datasets were analyzed in this study. The SemanticKITTI can be found at https://semantic-kitti.org/. The nuScenes can be found at https://www.nuscenes.org/.

## Appendix A

Specifically, given a LiDAR point cloud, each point $P_{n}\in R^{1\times 4}$ is represented by Cartesian coordinates $\left(P_{n}^{x},P_{n}^{y},P_{n}^{z}\right)$ and intensity $P_{n}^{I}$. Points are rasterized into a projection R(u,v) of size H×W based on azimuth angles θ and elevation-depression angles ϕ. The rasterization formula is as follows:

(A1) $$\left(\begin{array}{c} u_{n}\\ v_{n} \end{array}\right)=\left(\begin{array}{c} \frac{1}{2}\left[1-arctan\left(P_{n}^{y},P_{n}^{x}\right)\pi^{-1}\right]W\\ \left[1-\left(arcsin\left(P_{n}^{z},{\left(P_{n}^{d}\right)}^{-1}\right)+\varphi_{down}\right)f^{-1}\right]H \end{array}\right)$$

(A2) $$\left\{\begin{array}{c} P_{n}^{d}=\sqrt{{\left(P_{n}^{x}\right)}^{2}+{\left(P_{n}^{y}\right)}^{2}+{\left(P_{n}^{z}\right)}^{2}}\\ \varphi_{down}=\phi_{down}/180\times \pi \\ \varphi_{up}=\phi_{up}/180\times \pi \\ f=\left|\phi_{down}\right|+\left|\phi_{up}\right| \end{array}\right.$$

$\left(u_{n},v_{n}\right)$ represents the grid coordinates in the 2D image where point $P_{n}$ is projected. $\phi_{down}$ and $\phi_{up}$ represent the depression and elevation angles of the LiDAR device, respectively. (H,W) provides the range image resolution, and *H* aligns with the LiDAR laser beams.
